# Visualising Geophylogenies in Web Maps Using GeoJSON

**DOI:** 10.1371/currents.tol.8f3c6526c49b136b98ec28e00b570a1e

**Published:** 2015-06-23

**Authors:** Roderic Page

**Affiliations:** College of Medical, Veterinary & Life Sciences, Institute of Biodiversity, Animal Health and Comparative Medicine, University of Glasgow, Glasgow, UK

## Abstract

This article describes a simple tool to display geophylogenies on web maps including Google Maps and OpenStreetMap. The tool reads a NEXUS format file that includes geographic information, and outputs a GeoJSON format file that can be displayed in a web map application.

## Introduction

The increasing number of georeferenced sequences in GenBank 1 and the growth of DNA barcoding 2 means that the raw material to create geophylogenies 3 is readily available. However, constructing visualisations of phylogenies and geography together can be tedious. Several early efforts at visualising geophylogenies focussed on using existing GIS software 4, or tools such as Google Earth 5 ^,^ 6 ^,^ 7 . While the 3D visualisations enabled by Google Earth are engaging, it's not clear that they are easy to interpret. Another tool, GenGIS 12 ^,^ 13 , supports 2D visualisations where the phylogeny is drawn flat on the map, avoiding some of the problems of Google Earth visualisations. However, like Google Earth, GenGIS requires the user to download and install additional software on their computer.

By comparison, web maps such as Google Maps 15 and OpenStreetMap 16 are becoming ubiquitous and work in most modern web browsers. They support displaying user-supplied data, including geometrical information encoded in formats such as GeoJSON, making them a light weight alternative to 3D geophylogeny viewers. This paper describes a tool that makes use of the GeoJSON format and the capabilities of web maps to create quick and simple visualisations of geophylogenies.

## 2D layout of geophylogenies

The following discussion assumes that we have a phylogeny, and that for most (if not all) of the OTUs in that phylogeny are associated with a point locality for which we know the latitude and longitude.

In order to draw a geophylogeny on a web map we need to solve three problems. The first, relatively trivial problem is to place the the localities of the OTUs on the map (I shall refer to these as “occurrences”).

The second is to draw the phylogeny. Typically when drawing an evolutionary tree we compute *x* and *y* coordinates for a device where these coordinates have equal units and are linear in both horizontal and vertical dimensions, such as a computer screen or printer. In web maps coordinates are expressed in terms of latitude and longitude, and in the widely-used "web mercator" projection the *y*-axis (latitude) is non-linear. Furthermore, on a web map the user can zoom in and out, so pixel-based coordinates only make sense with respect to a particular zoom level.

**Web map tile:**
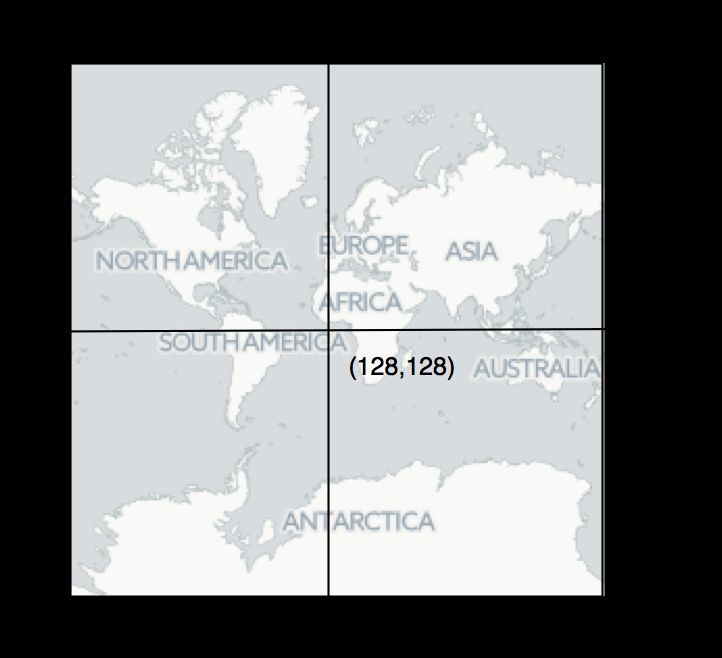
The single 256 × 256 pixel tile representing the globe a zoom level 0 showing the pixel coordinates for the top left corner, the centre (corresponding to longitude 0, latitude 0), and the bottom right. Tile image map tiles by CartoDB under CC-BY 3.0 license.

Web maps use “tiles” of a fixed size to represent the globe. Each tile is typically 256 × 256 pixels in size, and the number of tiles comprising a map is 2^*zoom*^ where *zoom* is the zoom level. At zoom level 0 the map comprises a single tile (Fig. 1), at zoom level 1 the map comprises 4 tiles, and zoom level 2 eight tiles, and so on. To accommodate the web mercator projection, we first compute a geographic bounding box for the tree based on the bounding box that encloses the occurrences, then offset that box so that so that the tree is drawn, say below, the occurrences. We can then convert the longitude λ and latitude Ω coordinates of the bounding box to pixels *x* and *y* at zoom level 0 using the following formulae:

\begin{equation*}x=128+\left( \lambda \frac{256}{360}  \right) \end{equation*}

\begin{equation*}y=\frac{1}{2}ln\left(\frac{1 + sin\phi}{1-sin\phi}  \right) \end{equation*}

Note that the maximum latitude that can be displayed in the web mercator projection is 85.051129° north and south. The tree drawing is then laid out within that bounding box, with the nodes positioned in terms of pixels. Once pixel coordinates have been computed for the whole tree, they are then converted back to latitude and longitude values:

\begin{equation*}\lambda = \left( x-128 \right) \frac{360}{256} \end{equation*}

\begin{equation*}\phi = \left( 2tan^{-1}\left( e^{\left( y-128 \right)\frac{-256}{2\pi }  }  \right)-\frac{1}{2} \pi    \right) \frac{180}{\pi } \end{equation*}

Expressing the tree in terms of latitude and longitude coordinates means that the rendering of the tree as the user zooms in and out is handled automatically by the web map application.

If we want to provide the user with a visual connection between each occurrence on the map and the location of the corresponding OTU in the phylogeny, we can draw a line connecting the two. These lines may criss-cross creating visual clutter, reducing this clutter is the third problem. To make the diagram more comprehensible, I adopt the approach used by GenGIS 12 ^,^ 13 to reorder the nodes in the tree to minimise the number of crossings 8. As an additional feature, if a taxon is represented by more than one occurrence, we can enclose the set of occurrences by a convex polygon to represent the range of that taxon.

Having computed a layout, we then need to render that on a web map. There are a number of different web maps available, each with their own API. Rather than tie the visualisation to a particular API, we can use a standardised output format, such as GeoJSON, to encode the layout, so that users can pick which web map they wish to use for the visualisation.

## GeoJSON

GeoJSON 17 is a format for encoding geographic data in JSON (JavaScript Object Notation). It includes various geometry types (such as Point, LineString, and Polygon), and is supported by a number of online mapping tools, including Google Maps 15 and Leaflet 18 . A GeoJSON document comprises a set of one or more features, each of which has a geometry and additional properties. Using the GeoJSON geometry types we can encode occurrences (Point), the tree (a set of LineString), and taxon distributions (Polygon) in GeoJSON, then have the entire visualisation rendered by the web application. The GeoJSON specification does not, by itself, include any information on how to display the objects encoded in a GeoJSON document (e.g., what colour to use for a line), but some informal standards have emerged, such as storing CSS styles as properties.

## Input format

In order to create the visualisation we also need a way to input a phylogeny and the geographic localities. The approach taken here is to use the NEXUS format 9 , and the GEOGRAPHIC datatype introduced by the Mesquite Cartographer package 14 . While some might argue that XML represents the future of phylogenetic file formats 10 , NEXUS is easy to manually edit and hence facilitates debugging and exploring the software. Given a set of OTUs, the tool expects a NEXUS file with a TREES block describing a tree, followed by a CHARACTERS block encoding the location of each OTU. Each OTU is typically a DNA sequence. Sets of sequence may belong to the same taxon (e.g., a species or a DNA barcode BIN 2 ). Following Mesquite, this information can be stored in an ALTTAXNAMES command in a NOTES block.

Figure 2 shows a NEXUS file for the widely used *Banza* example 11 ^,^ 19

**NEXUS file for Hawaiian Banza:**
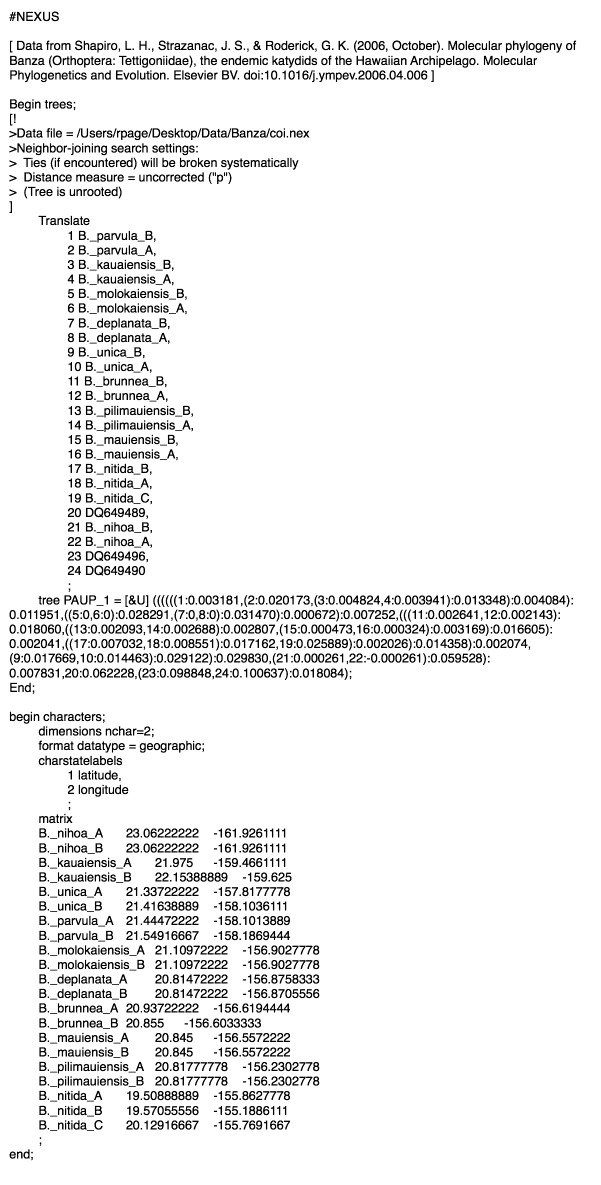
NEXUS file for Hawaiian Banza, with geographical data encoded in the CHARACTERS block.

## Implementation

I have implemented a NEXUS to GeoJSON converter using PHP. The code parses the NEXUS file, computes a bounding box based on the distribution of the OTUs, draws the tree, and exports the result in GeoJSON. The code is available on github https://github.com/rdmpage/geojson-phylogeny. Code for the examples in this article are available from https://github.com/rdmpage/geojson-phylogeny-manuscript/. A live demo can be explored at http://bionames.org/~rpage/geojson-phylogeny/ which includes examples of visualising geophylogenies using both Google Maps (Fig. 3) and Leaflet (Fig. 4).

**Geophylogeny for South American marsupial:**
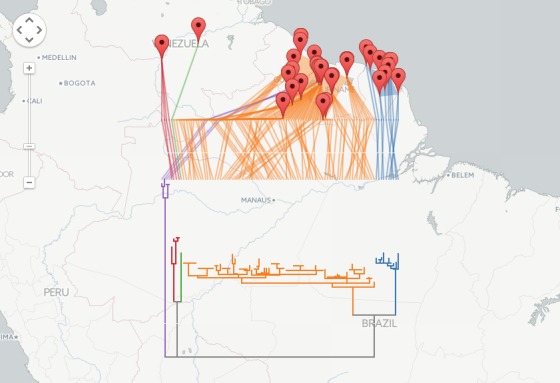
Geophylogeny for DNA barcodes for the marsupial Proechimys guyannensis, showing two distinct clusters that are geographically allopatric (data from BOLD, map tiles by CartoDB under CC-BY 3.0 license).

**Geophylogeny for Hawaiian katydids:**
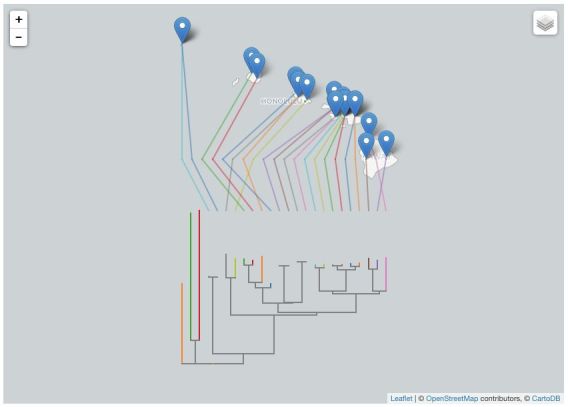
Geophylogeny for Hawaiian katydids (genus Banza) displayed using the Leaflet framework with map tiles by CartoDB under CC-BY 3.0 license.

## Discussion

At present the method described here requires a middle layer (written in PHP) that resides on a web server and converts the NEXUS file to GeoJSON. An obvious extension would be to port that code to Javascript and have the entire tool function within the web-browser client.

Although lacking some of the functionality of more specialised software such as GenGIS, an advantage of a web map-based tool is that it brings phylogenies into an environment already familiar to users of biodiversity data, such as the GBIF portal. Many users will have already encountered points on maps, and layers (e.g., of environmental data, or estimated species distributions). By representing phylogeny in GeoJSON we open the way for phylogenetic information to be incorporated into these maps.

Another reason GeoJSON is attractive is that because it is a JSON document it could be stored and indexed in a document database such as CouchDB 20 , which I've used elsewhere for taxonomic and phylogenetic data 21 . Hence we could imagine being able to quickly build a database of geophylogenies that can be queried both taxonomically and spatially. This would be one way to tackle the challenge of Kidd’s call for a “map of life”^3^.

## Competing interests

The authors have declared that no competing interests exist.

## References

[1] Marques AC, Maronna MM, Collins AG. Putting GenBank data on the map. Science. 2013 Sep 20;341(6152):1341. PubMed PMID:24052287. doi:10.1126/science.341.6152.1341-a 24052287 10.1126/science.341.6152.1341-a

[2] Ratnasingham S, Hebert PD. A DNA-based registry for all animal species: the barcode index number (BIN) system. PLoS One. 2013;8(7):e66213. PubMed PMID:23861743. doi:10.1371/journal.pone.0066213 23861743 10.1371/journal.pone.0066213 PMC3704603

[3] Kidd DM. Geophylogenies and the Map of Life. Syst Biol. 2010 Dec;59(6):741-52. PubMed PMID:20833950. doi:10.1093/sysbio/syq043 20833950 10.1093/sysbio/syq043

[4] Kidd DM, Liu X. geophylobuilder 1.0: an arcgis extension for creating 'geophylogenies'. Mol Ecol Resour. 2008 Jan;8(1):88-91. PubMed PMID:21585723. doi:10.1111/j.1471-8286.2007.01925.x 21585723 10.1111/j.1471-8286.2007.01925.x

[5] Arrigo N, Albert LP, Mickelson PG, Barker MS. Quantitative visualization of biological data in Google Earth using R2G2, an R CRAN package. Mol Ecol Resour. 2012 Nov;12(6):1177-9. PubMed PMID:22994899. doi:10.1111/1755-0998.12012 22994899 10.1111/1755-0998.12012

[6] Janies D, Hill AW, Guralnick R, Habib F, Waltari E, Wheeler WC. Genomic analysis and geographic visualization of the spread of avian influenza (H5N1). Syst Biol. 2007 Apr;56(2):321-9. PubMed PMID:17464886. 17464886 10.1080/10635150701266848

[7] Hill, A. W., & Guralnick, R. P. (2010, June 30). GeoPhylo: an online tool for developing visualizations of phylogenetic trees in geographic space. Ecography. doi:10.1111/j.1600-0587.2010.06312.x

[8] Barth, W., Mutzel, P., & Jünger, M. (2004). Simple and Efficient Bilayer Cross Counting. J. Graph Algorithms Appl. Journal of Graph Algorithms and Applications. doi:10.7155/jgaa.00088

[9] Maddison DR, Swofford DL, Maddison WP. NEXUS: an extensible file format for systematic information. Syst Biol. 1997 Dec;46(4):590-621. PubMed PMID:11975335. doi:10.1093/sysbio/46.4.590 11975335 10.1093/sysbio/46.4.590

[10] Cranston K, Harmon LJ, O'Leary MA, Lisle C. Best practices for data sharing in phylogenetic research. PLoS Curr. 2014 Jun 19;6. PubMed PMID:24987572. doi:0.1371/currents.tol.bf01eff4a6b60ca4825c69293dc59645 24987572 10.1371/currents.tol.bf01eff4a6b60ca4825c69293dc59645 PMC4073804

[11] Shapiro LH, Strazanac JS, Roderick GK. Molecular phylogeny of Banza (Orthoptera: Tettigoniidae), the endemic katydids of the Hawaiian Archipelago. Mol Phylogenet Evol. 2006 Oct;41(1):53-63. PubMed PMID:16781170. doi:10.1016/j.ympev.2006.04.006 16781170 10.1016/j.ympev.2006.04.006

[12] Parks DH, Mankowski T, Zangooei S, Porter MS, Armanini DG, Baird DJ, Langille MG, Beiko RG. GenGIS 2: geospatial analysis of traditional and genetic biodiversity, with new gradient algorithms and an extensible plugin framework. PLoS One. 2013;8(7):e69885. PubMed PMID:23922841. doi:10.1371/journal.pone.0069885 23922841 10.1371/journal.pone.0069885 PMC3726740

[13] Parks DH, Porter M, Churcher S, Wang S, Blouin C, Whalley J, Brooks S, Beiko RG. GenGIS: A geospatial information system for genomic data. Genome Res. 2009 Oct;19(10):1896-904. PubMed PMID:19635847. doi:10.1101/gr.095612.109 19635847 10.1101/gr.095612.109 PMC2765287

[14] Maddison, D.R., & W.P. Maddison. 2014. Cartographer, a Mesquite package for plotting geographic data. Version 1.41.

[15] Google Maps JavaScript API v3

[16] OpenStreetMap

[17] GeoJSON

[18] Leaflet

[19] Google Earth phylogenies

[20] CouchDB

[21] Page RD. BioNames: linking taxonomy, texts, and trees. PeerJ. 2013;1:e190. PubMed PMID:24244913. doi:10.7717/peerj.190 24244913 10.7717/peerj.190 PMC3817598

